# High Rate of Inappropriate Utilization of an Ophthalmic Emergency Department: A Prospective Analysis of Patient Perceptions and Contributing Factors

**DOI:** 10.3390/medicina61071163

**Published:** 2025-06-27

**Authors:** Helena Siegel, Vera Anna Widmer, Paola Kammrath Betancor, Daniel Böhringer, Thomas Reinhard

**Affiliations:** Eye Center, Medical Center—University of Freiburg, Faculty of Medicine, University of Freiburg, 79106 Freiburg im Breisgau, Germany

**Keywords:** ophthalmic emergency care, inappropriate utilization, patient perception of urgency, ambulatory care access, triage, eye diseases, healthcare-seeking behavior

## Abstract

*Background and Objectives*: In Germany, access to medical care is often hindered by long wait times for specialist appointments and emergency department care. Inappropriate utilization of emergency services further exacerbates delays for truly urgent cases. To evaluate the utilization of the statutory ophthalmic emergency service in Freiburg and identify patient- and system-level factors contributing to inappropriate use. *Materials and Methods*: A paper-based, anonymous questionnaire was distributed to patients attending the ophthalmologic emergency practice (*Notfallpraxis*) of the Association of Statutory Health Insurance Physicians (*Kassenärztliche Vereinigung*), which is located within the premises of the Eye Center of the University Hospital Freiburg, Germany, at selected periods between July and September 2020, alongside a short physician assessment. Standardized instruments were used to assess symptom severity, urgency perception, and healthcare-seeking behavior. Statistical analyses were performed using R and Excel. *Results*: A total of 157 questionnaires were included (response rate: 63%). Most visits occurred on weekends (47%) and before 10 p.m. (83%). While 68% of patients believed their symptoms required same-day treatment, physicians assessed only 30% of cases as clinically urgent. A total of 60% of patients did not attempt to contact an outpatient ophthalmologist beforehand, and only 38% reported having a regular ophthalmologist. Patients’ perceived urgency was significantly associated with symptom severity and older age, whereas physician-assessed urgency was strongly linked to symptom duration. *Conclusions*: A substantial proportion of ophthalmic emergency visits in Freiburg are for non-urgent conditions. These findings underscore the need for improved coordination with outpatient care providers, better patient education, and structural reforms to reduce inappropriate utilization and ensure timely access for truly urgent cases.

## 1. Introduction

Emergency departments in many healthcare systems are increasingly challenged by patients presenting with non-urgent ophthalmologic conditions [1,2,3,4,5,6,7]. In Germany, as in other countries, long wait times for outpatient appointments and limited access to regular eye care—especially during evenings and weekends—may contribute to patients seeking care in emergency settings, even when their symptoms do not require immediate medical attention.

This pattern not only places unnecessary strain on emergency services but also risks delaying timely treatment for patients with acute, vision-threatening conditions [8]. A growing body of literature suggests that subjective urgency, health-related anxiety, and structural barriers such as limited appointment availability can all influence patients’ decision to bypass regular outpatient care. Notably, physicians’ clinical assessments of urgency often diverge significantly from patients’ perceived need for immediate care.

To address issues of accessibility, the German government introduced the Healthcare Strengthening Act in 2015, mandating the creation of centralized regional appointment service centers (*Terminservicestellen*). These services aim to facilitate access to outpatient specialist care—such as ophthalmology—within a four-week window, even without referral [9]. However, the continued high rate of non-urgent presentations to ophthalmologic emergency services suggests that such organizational reforms alone may not adequately resolve the underlying drivers of emergency service utilization.

To date, only a limited number of studies have systematically compared patient-reported motivations, perceptions of urgency, and healthcare-seeking behavior with physicians’ clinical evaluations in the context of ophthalmologic emergencies. Understanding this discrepancy is essential for improving triage processes, patient education, and service design.

### Aim of the Study

The present study is a prospective, questionnaire-based field investigation conducted at the emergency ophthalmology service of the Eye Center of the University Hospital Freiburg, Germany. Its objectives are to

- Explore patients’ motivations for utilizing emergency services and their subjective assessment of urgency;
- Identify structural and psychological barriers to accessing regular outpatient eye care;
- Compare patient-reported urgency with the urgency as assessed by treating ophthalmologists.

By capturing both patient and physician perspectives, this study aims to contribute to a more nuanced understanding of the factors underlying emergency service overuse in ophthalmology and to inform strategies for improving access, triage, and care coordination.

## 2. Materials and Methods

### 2.1. Study Design and Setting

The present study is a prospective, questionnaire-based field investigation conducted at the ophthalmologic emergency practice (*Notfallpraxis*) of the Association of Statutory Health Insurance Physicians (*Kassenärztliche Vereinigung*), which is located within the premises of the Eye Center of the University Hospital Freiburg, Germany. The study focused on patients who presented to the walk-in ophthalmic emergency service.

The survey was conducted during four randomly selected one-week sampling periods within a two-month time frame (27 July to 2 August, 10 to 16 August, 24 to 30 August, and 7 to 13 September 2020) to ensure coverage across various seasonal and structural conditions such as holidays and variable patient volumes. Each week included both weekday and weekend shifts to ensure temporal representativeness.

All adult patients who attended the ophthalmologic emergency practice of the Association of Statutory Health Insurance Physicians during these predefined periods and experienced a wait time before being seen were invited to participate. Patients seen immediately (e.g., in low-load periods or due to urgent triage) or directly admitted were excluded. In the case of minors, accompanying guardians were invited to complete the questionnaire.

A formal sample size calculation was not performed, as this was an exploratory study aimed at descriptive and comparative analysis. Nevertheless, the achieved sample size of 157 patients (response rate: 63%) provides sufficient statistical power to detect moderate associations in bivariate and multivariate analyses.

### 2.2. Questionnaire Structure and Administration

The questionnaire was paper-based and self-administered by the patients while waiting for their consultation. It included clear instructions on the cover page and was handed out accompanied by a sealable envelope. Participation was voluntary and anonymous. Patients could indicate non-participation by returning a blank questionnaire. The questionnaire was returned in a sealed envelope, and the treating physician subsequently rated the clinical urgency on the back side of the envelope.

The questionnaire was adapted from a validated instrument developed by Frick et al. [10] for assessing patient decision making in emergency departments (see Files S1 and S2 in Supplementary Materials). The content was reviewed by medical staff and laypersons and tailored to ophthalmologic complaints based on literature review, patient data analysis, focus group interviews with attending physicians, and a preliminary pilot study.

The survey instrument comprised two sections. The first focused on the patient’s symptoms. Patients were asked to indicate their main reason for visiting the emergency service by selecting one predefined symptom from the following list: pain, swelling, trauma or injury, redness, visual impairment, or diplopia. Only one option was permitted. Furthermore, they were asked to assess their perceived urgency of care. The second section addressed healthcare access, prior contact with outpatient services, and knowledge of alternative care options. Basic demographic data (age, gender) were collected at the end of the questionnaire.

### 2.3. Physician Urgency Assessment

To assess the discrepancy between perceived and clinically assessed urgency, physicians completed two short items regarding the medical urgency of the case. These assessments were matched with patients’ self-ratings for further analysis.

### 2.4. Statistical Analysis

Statistical analysis was performed using R 4.0.3 and 4.4.2 (R Foundation for Statistical Computing, Vienna, Austria) and Microsoft Excel 2016 (Microsoft Corporation, Redmond, WA, USA). Continuous data are presented as mean ± standard deviation and categorical variables as absolute and relative frequencies. Chi-square tests were used to compare proportions across groups. For metric data, *t*-tests or one-way ANOVA were applied, as appropriate.

To identify factors influencing urgency perception and utilization behavior, logistic regression models were fitted. Associations between continuous and ordinal variables were examined using Pearson or Spearman correlation coefficients, depending on the data scale. A *p*-value < 0.05 was considered statistically significant. No correction for multiple testing was applied.

### 2.5. Ethical Considerations and Data Protection

All data were anonymized, and participation was voluntary. Due to the anonymous and non-interventional nature, ethical approval for the study was granted by the Ethics Committee of the University of Freiburg, Germany (application number: 372/19). The Association of Statutory Health Insurance Physicians of Southern Baden (Kassenärztliche Vereinigung) in Germany provided approval for data collection and analysis.

The anonymized datasets used for this study are available upon reasonable request.

### 2.6. Generative AI Disclosure

Generative AI tools (ChatGPT by OpenAI, accessed March 2025, version GPT-4) were used to assist in language refinement. No AI tools were used for data analysis, study design, or interpretation.

## 3. Results

### 3.1. Patient Population

Of the 250 questionnaires distributed, 157 were included in the final analysis, yielding a response rate of 63%. Nine were returned blank, and ten lacked physician input. All partially completed questionnaires were retained, resulting in varying denominators across individual items.

The mean patient age was 39 ± 20 years (median: 37; range: 1–89), with 41% of participants aged between 21 and 40 years. Among the 140 respondents who reported their gender, 42.1% (*n* = 59) were female. The majority of patients presented on weekends (62%, *n* = 97), and 83% (*n* = 131) were seen before 10 p.m.

### 3.2. Symptom Characteristics

The mean reported symptom severity was 5.55 ± 2.54 on a 0–10 visual analog scale. In 67% of cases (*n* = 105), the symptoms were of new onset. Chronic symptoms were reported by 6% (*n* = 10), and 18% (*n* = 28) described them as recurrent. Nearly half of the respondents (48%, *n* = 75) stated that symptom onset occurred on the day of presentation. a total of 17% (*n* = 27) had previously visited an emergency service within the past six months.

### 3.3. Perceived vs. Clinical Urgency

A total of 68% (*n* = 107) of patients believed that their symptoms required treatment on the same day, with 27% (*n* = 43) considering immediate treatment necessary. The perceived urgency significantly correlated with reported symptom severity (ANOVA, *p* < 0.01, *n* = 143; Table 1).

Physicians consistently rated trauma and visual loss as more urgent than patients did, whereas symptoms such as redness, pain, and swelling were associated with notably lower clinical urgency (see Table A1 in Appendix A). Patients’ median urgency rating was 4 (“same-day treatment”), whereas physicians’ median rating was 3 (“within a few days”), with a mean of 2.73. The correlation between patient and physician urgency ratings was weak (Spearman ρ = −0.06; Chi-square *p* = 0.46, *n* = 144).

Agreement between patient and physician assessments was found in only 21% (*n* = 30) of cases. In contrast, 67% (*n* = 94) of patients overestimated and 5% (*n* = 7) underestimated the clinical urgency (Table 2).

**Figure 1:**
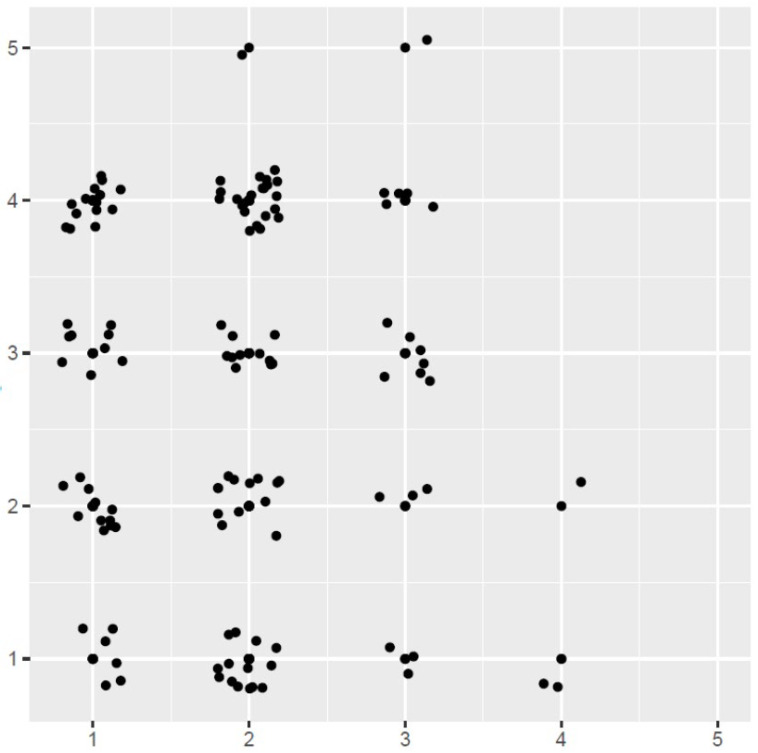
Correlation between patient- and physician-rated urgency. Scatterplot showing relationship between patient-perceived and physician-assessed urgency (1 = immediate, 2 = within 1 h, 3 = within 1–2 days, 4 = within 1 week, and 5 = non-urgent/months). Jittering was applied to improve visibility.

Figure 1 illustrates the distribution of perceived vs. physician-assessed urgency. The widespread scatter of data points visually confirms the lack of correlation between patient and physician assessments.

### 3.4. Determinants of Urgency Assessment

**Figure 2:**
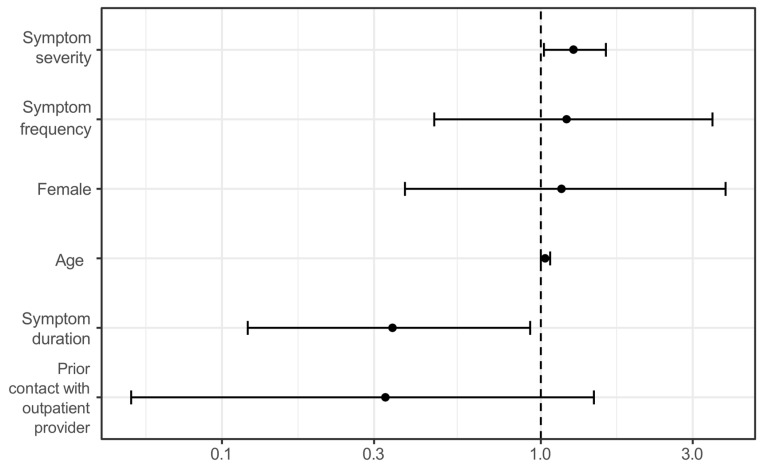
Odds ratios for high patient-perceived urgency. Multivariable logistic regression analysis of patient characteristics associated with high perceived urgency (score 1–2 on a 5-point scale). Odds ratios (ORs) and 95% confidence intervals (CIs) are shown for each predictor. Significant associations were observed for increasing age, higher symptom severity, and shorter symptom duration. Prior contact with an outpatient provider showed a non-significant trend toward lower perceived urgency.

Multivariate logistic regression revealed distinct patterns in urgency assessment between patients and physicians. Patient-perceived urgency was significantly associated with higher age (OR = 1.033; 95% CI: 1.002–1.070), while gender showed no significant effect (OR = 1.162; 95% CI: 0.375–3.799). Prior contact with an outpatient physician showed a non-significant trend toward lower urgency (OR = 0.326; 95% CI: 0.052–1.469). Symptom severity was a significant predictor of higher perceived urgency (OR = 1.268; 95% CI: 1.024–1.601), while frequency of complaints was not. Shorter symptom duration was associated with higher perceived urgency (OR = 0.343; 95% CI: 0.120–0.926) (Figure 2).

Logistic regression model indicating factors significantly associated with higher urgency ratings by patients (score 1–2 on a 5-point scale).

**Figure 3:**
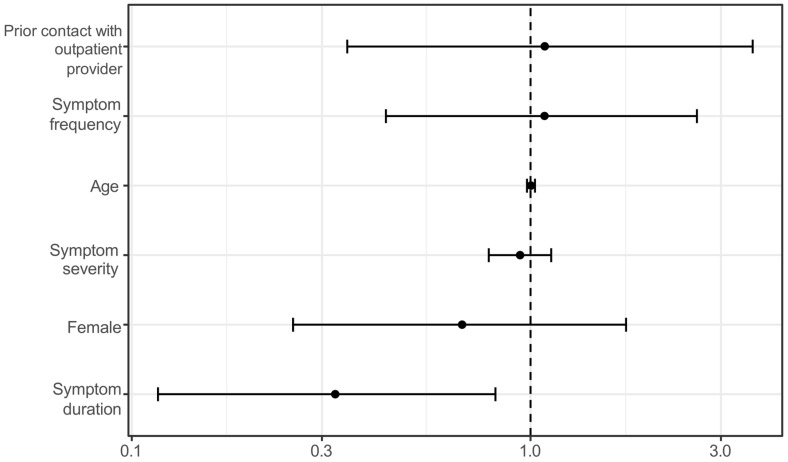
Odds ratios for high physician-assessed urgency. Multivariable logistic regression analysis of patient-related factors associated with high physician-rated urgency. Odds ratios (ORs) and 95% confidence intervals (CIs) are presented. Only shorter symptom duration was significantly associated with higher clinical urgency ratings. Other variables, including age, gender, symptom severity, frequency, and prior contact with a physician, were not significantly associated.

In contrast, physician-rated urgency was not significantly influenced by age (OR = 1.002; 95% CI: 0.979–1.025), gender (OR = 0.673; 95% CI: 0.254–1.735), symptom severity (OR = 0.941; 95% CI: 0.785–1.126), frequency (OR = 1.084; 95% CI: 0.434–2.612), or prior contact (OR = 1.086; 95% CI: 0.347–3.601). However, longer symptom duration significantly predicted lower urgency (OR = 0.324; 95% CI: 0.116–0.816) (Figure 3).

Logistic regression model showing that only symptom duration significantly predicted higher urgency ratings by physicians.

### 3.5. Additional Influencing Factors

A total of 62% (*n* = 82) of patients expressed health-related concerns, which were significantly associated with higher perceived urgency (*p* < 0.01, *n* = 115). Professional or family obligations were infrequently mentioned as reasons for emergency presentation (17%, *n* = 22).

### 3.6. Access to Care and Knowledge of Alternatives

Most patients (80%, *n* = 126) reported having a regular general practitioner, while only 38% (*n* = 60) had a regular ophthalmologist. A majority presented without having contacted a physician beforehand (73% on weekends; 66% on weekdays; Chi-square *p* = 0.40). Among those who had made contact (23%, *n* = 37), 39% (*n* = 22) were referred to the hospital.

Women were significantly more likely than men to report having a regular ophthalmologist (60% vs. 31%, *p* < 0.01) and were more likely to have contacted them before presentation (36% vs. 20%, *p* = 0.04). Patients seen during the week were less likely to report a regular ophthalmologist than those seen on weekends (64% vs. 40%, *p* = 0.04). Presentation during late hours was also significantly associated with the absence of a regular ophthalmologist (88% vs. 44%, *p* = 0.02).

Despite lacking prior contact with a provider, 38% (*n* = 50) of patients believed a regular outpatient clinic would have sufficed. Another 32% (*n* = 43) were unsure, and 16% (*n* = 21) viewed emergency care as necessary. A total of 65% (*n* = 86) assumed that outpatient clinics were closed at symptom onset, and only 29% (*n* = 39) believed an alternative ophthalmologic care option would have been available.

Patients who rated their condition as highly urgent were less likely to have a regular ophthalmologist (approximately 50% vs. 30%, *p* = 0.20) and less likely to believe that outpatient care would have sufficed (*p* = 0.10). However, no significant association was found between access to outpatient care and perceived urgency (*p* = 0.70).

## 4. Discussion

### 4.1. Inappropriate Utilization of Ophthalmic Emergency Services in Freiburg

The primary aim of this study was to evaluate the current utilization of the statutory ophthalmic emergency service in Freiburg and to identify factors contributing to its inappropriate utilization. The findings suggest that a significant proportion of visits to the emergency service are non-urgent, primarily driven by limited patient knowledge and structural barriers within the healthcare system.

More than two-thirds of surveyed patients believed their symptoms required treatment on the same day, whereas physicians deemed only 30% of cases to require urgent intervention. The discrepancy between patients’ subjective urgency and physicians’ clinical assessment is a key factor contributing to inappropriate utilization.

### 4.2. Factors Contributing to Inappropriate Utilization

The underlying reasons are multifaceted. Patients tend to base their urgency assessment on the subjective intensity of symptoms. Greater symptom severity was significantly associated with higher perceived urgency (OR = 1.268, 95% CI: 1.024–1.601), while this factor did not influence physicians’ assessments. Age also played a role in perceived urgency: older patients were more likely to rate their condition as urgent (OR = 1.033, 95% CI: 1.002–1.070), possibly reflecting heightened health anxiety or a greater need for reassurance.

In contrast, physicians based their urgency assessments primarily on the duration of symptoms. Longer symptom duration was significantly associated with lower clinical urgency (OR = 0.324, 95% CI: 0.116–0.816). This clinical approach is consistent with findings from other countries. For example, in the USA, Sridhar et al. [4] reported that 73.9% of patients presenting with symptoms lasting less than one week were classified as urgent, compared to only 36.3% for those with longer symptom durations (*p* < 0.001).

The outpatient sector plays a central role in the context of inappropriate utilization. A substantial proportion of patients reported no prior contact with an office-based ophthalmologist before visiting the emergency service, highlighting both weak integration into the outpatient care network and underuse of available specialist services. Reasons for this may include unsuccessful attempts to schedule appointments, a lack of continuous ophthalmologic follow-up, or the assumption that practices were closed at the time of symptom onset—particularly during evenings or weekends. Many patients also chose to attend the emergency unit based on expectations of higher-quality care, more comprehensive diagnostics, or quicker access to advanced procedures. A similar finding was reported by Sempere-Selva et al. [11] in Spain, where expectations of higher clinical quality in hospitals vs. primary care were among the main reasons cited for emergency department visits—indicating that such perceptions are not limited to ophthalmology or national boundaries.

Inadequate knowledge about appropriate emergency care pathways has also been reported internationally and across medical specialties [6,12]. This may lead to inappropriate utilization, resulting in long wait times [8,13] and delayed access for time-critical emergencies. In turn, this could negatively impact outcomes for patients in true need of urgent care.

### 4.3. Comparison with International Studies

Our findings are consistent with a wide range of international studies documenting high rates of non-urgent visits to ophthalmic emergency services.

In the UK, a few investigations have highlighted this issue. Kheterpal, Perry, and McDonnell [1] reported that between 50% and 70% of ophthalmic emergency visits were non-acute. Hau et al. [2] found that only 30.6% of patients were classified as urgent in a British emergency eye department, while 37.5% could have been managed by specially trained general practitioners or optometrists.

Outside the UK, similar patterns have emerged. In Dublin, Ireland, 60–70% of emergency visits were non-urgent, and 54% of patients presented without a referral [8]. In Brazil, a retrospective study in São Paulo showed that 69% of cases presenting to the ophthalmic emergency unit could have been managed in primary or secondary care settings [3]; a more recent study also conducted in São Paulo [7] even documented a significant increase of 39.2% in visits to an ophthalmic emergency department between 2009 and 2019, with non-urgent consultations also predominating.

A national analysis from Taiwan [5] indicated that only 48.2% of emergency consultations were classified as urgent, while 30.9% were non-urgent. Sridhar et al. [4] found similar results in the USA, reporting 35.8% of visits as non-acute.

### 4.4. Contrasting the German Healthcare System with International Settings

Comparison with international data highlights how structural differences in healthcare systems affect utilization patterns in ophthalmic emergency care. In countries such as Brazil, Taiwan, and the USA, emergency departments often serve as the first point of contact due to limited access to outpatient specialists or high out-of-pocket costs [4,5,7].

In contrast, Germany’s dual healthcare system provides broad access to office-based ophthalmologists, who serve as the primary providers of outpatient eye care. Emergency services are intended to serve patients with acute, potentially sight-threatening conditions. Nonetheless, a substantial number of patients with non-urgent issues continue to seek care at emergency departments, such as the ophthalmic emergency service housed at the University Hospital in Freiburg. This appears to be driven by inadequate access to outpatient appointments, particularly outside of regular office hours, failed appointment attempts, and subjective perceptions of urgency.

The German setting thus reflects a complex tension: while a robust outpatient system exists, barriers such as accessibility, health literacy, and patient expectations continue to hinder effective care coordination. For example, statutory health-insured patients continue to face significantly longer wait times for specialist appointments compared to privately insured individuals, a disparity that has persisted despite health policy interventions [14]. Geographic maldistribution further exacerbates accessibility gaps, with rural regions showing lower ophthalmologist density and limited availability of telemedical services [15]. Moreover, population-level health literacy remains limited in Germany, with nearly half of adults exhibiting significant difficulties in accessing, understanding, appraising, or applying health-related information, a factor known to increase inappropriate emergency department utilization [16]. Additionally, qualitative data indicate that uncertainties regarding responsibilities within the healthcare system and difficulties in securing timely outpatient care appointments contribute to patients’ decisions to seek help at emergency departments, even for non-urgent conditions, particularly when immediate diagnostic clarification is expected [17]. In contrast, countries like Brazil, Taiwan, or the USA experience emergency department overuse for different systemic reasons, often due to underdeveloped or inaccessible outpatient infrastructures and higher out-of-pocket costs. Nonetheless, these international examples also highlight how structural constraints, irrespective of system design, shape patient navigation behavior and healthcare-seeking patterns.

### 4.5. Limitations and Contextual Considerations

This study has several limitations that should be considered when interpreting the results. First, participation in the survey was voluntary and limited to patients who experienced a waiting period before being seen. This may have introduced response bias, as individuals with heightened concern, stronger opinions, or greater willingness to engage may have been more likely to participate. The findings may therefore not fully reflect the perceptions of all patients presenting to the emergency service.

Second, the study was conducted at a single urban university hospital in southern Germany. As such, generalizability to rural areas or other healthcare systems with different outpatient infrastructures may be limited.

Third, we did not collect standardized diagnostic data or follow-up outcomes. As a result, it is not possible to determine whether patient- or physician-rated urgency corresponded to actual disease severity or clinical benefit. This limits the conclusions that can be drawn regarding the appropriateness of emergency care utilization. In particular, the lack of diagnostic coding precluded analysis of whether patients with high symptom intensity also suffered from clinically severe or sight-threatening conditions. While this limits interpretation, physician-assessed urgency can be regarded as a pragmatic surrogate for disease severity, given that it was based on clinical examinations by experienced ophthalmologists. Our data suggest that symptoms commonly perceived as alarming by patients, such as pain or redness, were often rated as displaying low urgency by clinicians, whereas symptoms such as visual loss or trauma, which may initially be less painful, were rated as clinically urgent. This highlights the known discrepancy between symptom burden and medical severity in ophthalmology, as seen in conditions like superficial corneal erosions compared to central retinal vein occlusion. Future studies should incorporate structured diagnostic data to enable such correlations.

Fourth, although the survey captured contextual indicators such as regular access to ophthalmologists or general practitioners and attempted prior contact, it did not assess more nuanced factors like education level, health literacy, or previous outpatient experiences. These could substantially influence patients’ urgency perception and healthcare navigation.

Lastly, although most outpatient ophthalmology practices in Germany had resumed services by mid-2020, some may still have operated with reduced scheduling flexibility or capacity due to ongoing COVID-19 hygiene protocols. Additionally, pandemic-related anxiety may have affected healthcare-seeking behavior. These contextual factors may have contributed to increased emergency service utilization during the study period.

## 5. Conclusions

This study demonstrates that a considerable proportion of ophthalmic emergency visits are for non-urgent conditions. The inappropriate utilization of emergency care is largely driven by subjective perceptions of urgency, limited patient knowledge, and structural barriers to accessing routine outpatient ophthalmologic services. While patients—especially older individuals—tend to assess urgency based on symptom severity, physicians rely primarily on the duration of symptoms when determining clinical necessity.

Reducing this burden on emergency services requires multifaceted interventions, i.e., improved patient education, better access to routine eye care, and optimized triage systems. Public health campaigns, training for healthcare providers, and well-integrated referral pathways between outpatient and emergency care represent essential strategies for ensuring that ophthalmic emergency services remain accessible for truly urgent cases.

## Figures and Tables

**Table 1:** Perceived urgency and symptom severity.

Perceived Urgency (By Patient)	Mean/Median/Max Symptom Severity
Immediate	5/7/9
Within 1 h	4/5/7
Within a few days	3/4.5/6
Within weeks	2/4/4.5
Within months	10/10/10

This table shows the relationship between patients’ perceived treatment urgency and the severity of their symptoms as rated on a visual analog scale (0–10). The differences between the groups were statistically significant (one-way ANOVA, *p* < 0.001).

**Table 2:** Comparison of patient- and physician-rated urgency.

Patient-Rated Urgency	Physician-Rated Urgency (Mean)	Physician-Rated Urgency (Median)
Immediate (5)	2.78	Within a few days (3)
Within 1 h (4)	2.72	Within a few days (3)
Within 1–2 days (3)	2.89	Within a few days (3)
Within weeks (2)	1.33	Within months (1)
Within months (1)	Not applicable	Not applicable

This table compares patients’ perceived urgency with physicians’ mean and median urgency assessments. It illustrates the systematic overestimation of urgency by patients, particularly for symptoms perceived as requiring immediate or same-day care.

## Data Availability

The datasets generated and analyzed during the current study are not publicly available due to patient confidentiality and institutional data protection policies. Reasonable requests for access to the anonymized data should be directed to the corresponding author.

## Supplementary Materials

The following supporting information can be downloaded at: https://www.mdpi.com/article/10.3390/medicina61071163/s1, Supplementary File S1: Original German-Language Patient Questionnaire Used in the Study, Including Physician Evaluation Section; Supplementary File S2: Patient Questionnaire (English Translation).

## Abbreviations

The following abbreviations are used in this manuscript:

OR	Odds Ratio
CI	Confidence Interval
ANOVA	Analysis of Variance
AI	Artificial Intelligence

## Appendix A

**Table A1:** Physician-rated urgency by symptom.

Symptom	Symptom Present—Mean/Median	Symptom Absent—Mean/Median	Interpretation
Pain	2.59/3	2.88/3	Slightly lower urgency when present.
Trauma	3.14/4	2.63/3	Markedly higher urgency when present.
Vision loss	3.15/3	2.66/3	Higher urgency when present.
Swelling	2.33/2	2.90/3	Lower urgency when present.
Redness	2.34/2	3.07/3	Lower urgency when present.
Double vision	2.00/2	2.77/3	Higher urgency, limited cases.

This table summarizes the physicians’ urgency assessments (mean and median values) based on the presence or absence of specific symptoms. Urgency was rated on a 5-point scale (1 = elective; 5 = emergency).
