# Supplementary material for: High Rate of Inappropriate Utilization of an Ophthalmic Emergency Department: A Prospective Analysis of Patient Perceptions and Contributing Factors
Source: Medicina (Kaunas). 2025 Jun 27;61(7):1163. doi: 10.3390/medicina61071163 (PMC12300961; doi:10.3390/medicina61071163)
Supplement: Supplementary file 1 [file medicina-61-01163-s001.zip › S1_Questionnaire_german.pdf]

Sehr geehrte Patientin,

Sehr geehrter Patient,

wir bitten Sie, die folgenden Fragen zu beantworten, weil wir mehr über Ihre Gründe für das Aufsuchen der augenärztlichen Notfallpraxis lernen wollen.

Der Fragebogen besteht aus 12 Fragen, die Sie in wenigen Minuten ausfüllen können.

Sie bleiben bei der Befragung vollkommen anonym.

Bitte so markieren: ☐ ☒ ☐ ☐

Bitte verwenden Sie einen Kugelschreiber.

Ihre Angaben dürfen von Ihrem behandelnden Arzt nicht eingesehen werden. Bitte überreichen Sie den Fragebogen daher im **verschlossenen** Umschlag.

Es entstehen Ihnen **keine Nachteile**, auch dann nicht, wenn Sie nicht teilnehmen. In diesem Fall können Sie den Fragebogen unausgefüllt im verschlossenen Umschlag abgeben oder den Fragebogen samt Umschlag wegwerfen.

Vielen Dank, dass Sie den Fragebogen ausfüllen!

Beschreibung Ihrer aktuellen gesundheitlichen Beschwerden (bitte immer nur ein Kreuz)

1. Mit welcher Hauptbeschwerde bzw. welchem Anlass stellen Sie sich heute in der Nofallpraxis vor?

Kreuzen Sie einen Grund an				Kreuzen Sie eine Lokalisation an	
Schmerzen	☐	Schwellung	☐	Augapfel	☐
Unfall/Verletzung	☐	Rötung	☐	Lider	☐
Sehverschlechterung	☐	Doppelsehen	☐		

2. Innerhalb welcher Zeitspanne müssen Sie Ihrer Ansicht nach medizinisch behandelt werden?

Sofort	1 Stunde	1-2 Tage	1 Woche	1 Monat oder länger
☐	☐	☐	☐	☐

3. Wie häufig treten Ihre aktuellen Beschwerden auf?

Zum ersten Mal ☐ Immer wieder ☐ Sind dauerhaft ☐

4. Wie stark sind Ihre aktuellen Beschwerden?

Von 0 (überhaupt nicht stark) bis 10 (extrem stark)

0	1	2	3	4	5	6	7	8	9	10
☐	☐	☐	☐	☐	☐	☐	☐	☐	☐	☐

5. Seit wann bestehen Ihre Beschwerden, mit denen Sie sich heute vorstellen?

Seit Heute	Seit Tagen	Seit Wochen	Seit Monaten
☐	☐	☐	☐

6. Wie würden Sie die Behandlung in der Notfallpraxis gegenüber der Behandlung in einer "normalen" Praxis einschätzen?

**Notfallpraxis**	**schlechter**	**gleich**	**besser**
Wartezeit	☐	☐	☐
Qualität der medizinischen Versorgung	☐	☐	☐

Gesamte Behandlungsdauer	☐	☐	☐
Diagnostische/ therapeutische Möglichkeiten	☐	☐	☐
Versorgung rund um die Uhr	☐	☐	☐
Abklärung unklarer Beschwerden	☐	☐	☐

Beschreibung Ihrer gesundheitlichen Versorgung

7. Wie oft waren Sie in den letzten 6 Monaten in einer Notfallpraxis (nicht nur für die Augen)?

Kein mal ☐ 1 mal ☐ 2-3 mal ☐ 4 mal und mehr ☐

8. Haben Sie einen Hausarzt?

Ja ☐ Nein ☐ Ja, aber nicht in der Region ☐

9. Haben Sie einen Augenarzt?

Ja ☐ Nein ☐ Ja, aber nicht in der Region ☐

10. Haben Sie aufgrund Ihrer aktuellen Beschwerden eine Arztpraxis kontaktiert? (Zu einem Kontakt zählen der Besuch einer Praxis oder ein Anruf in einer Praxis)

Ja ☐ (Fahren Sie bitte mit den Fragen in 11 A fort)

Nein ☐ (Fahren Sie bitte mit den Fragen in 11 B fort, **umblättern**)

A: Wenn Sie eine Praxis kontaktiert haben

11. Bitte beantworten Sie folgende Fragen nur, wenn Sie in Frage 10 „ja“ angekreuzt haben

1. Konnten Sie dem Praxisteam Ihre Beschwerden mitteilen?

   Ja ☐ Nein ☐

2. Hatten Sie einen Arztkontakt? (z.B. Gespräch, Untersuchung)

   Ja ☐ Nein ☐

3. War der zugeteilte Untersuchungstermin Ihrer Meinung nach zu spät?

   Ja ☐ Nein ☐

4. Hat Ihnen die Praxis eine Behandlung am Universitätsklinikum empfohlen?

   Ja ☐ Nein ☐

B: Wenn Sie keine Praxis kontaktiert haben

11. Bitte beantworten Sie folgende Fragen nur, wenn Sie in Frage 10 „Nein“ angekreuzt haben

1. Kennen Sie die Telefonnummer "116117" für den ärztlichen Bereitschaftsdienst?

   Ja ☐ Nein ☐

2. Hatten Sie versucht, eine "normale" Praxis zu kontaktieren?

   Ja ☐ Nein ☐

3. Hatten Sie vermutet, diese Praxis könnte aktuell geschlossen sein?

   Ja ☐ Nein ☐

4. Ist eine "normale" Praxis für die Behandlung Ihres Problems Ihrer Meinung nach ausreichend?

   Ja ☐ Nein ☐ Weiß nicht ☐

5. Steht Ihnen eine "normale" Augenarzt-Praxis zur Verfügung?

   Ja ☐ Nein ☐ Weiß nicht ☐

6. Konnten Sie aus beruflichen oder familiären Gründen eine "normale" Praxis nicht in Anspruch nehmen?

   Ja ☐ Nein ☐

7. Sind Sie aufgrund Ihrer Beschwerden sehr beunruhigt?

   Ja ☐ Nein ☐

8. Haben Sie das Universitätsklinikum aufgesucht, weil Sie hier bereits behandelt wurden?

   Ja ☐ Nein ☐

Angaben zu Ihrer Person

12. Bitte geben Sie Ihr Alter und Geschlecht an:

Alter (Lebensjahr) ___________ Geschlecht (m/w/d) _____

Von der Ärztin / vom Arzt auszufüllen

Umschlag nicht öffnen!

Bitte so markieren: ☐ ☒ ☐ ☐

1. Wie dringlich war die Behandlung?

- ☐ Elektiv (ausreichend innerhalb von Monaten)
- ☐ Dringlich (ausreichend innerhalb von Wochen)
- ☐ Hochdringlich (ausreichend innerhalb weniger Tage)
- ☐ Akut (musste heute behandelt werden)
- ☐ Notfall (musste sofort behandelt werden, keine Wartezeit)

2. Schätzen Sie folgende Parameter ein

**Schmerz**	**Rötung**	**Visusverlust**	**Bulbuseröffnungs-risiko**
Nicht vorhanden ☐	Nicht vorhanden ☐	Nicht vorhanden ☐	Nicht vorhanden ☐
Mittel ☐	Mittel ☐	Mittel ☐	Mittel ☐
Schwer ☐	Schwer ☐	Schwer ☐	Schwer ☐

3. Zeitpunkt der Untersuchung

1. Wochenende

   ☐ Ja ☐ Nein

2. Später als 22 Uhr

   ☐ Ja ☐ Nein
