# Supplementary material for: High Rate of Inappropriate Utilization of an Ophthalmic Emergency Department: A Prospective Analysis of Patient Perceptions and Contributing Factors
Source: Medicina (Kaunas). 2025 Jun 27;61(7):1163. doi: 10.3390/medicina61071163 (PMC12300961; doi:10.3390/medicina61071163)
Supplement: Supplementary file 1 [file medicina-61-01163-s001.zip › S2_Questionnaire_english_translation.pdf]

Dear Patient,

We kindly ask you to answer the following questions to help us better understand your reasons for visiting the ophthalmology emergency department.

The questionnaire contains 12 questions and takes only a few minutes to complete.

Your responses are completely anonymous.

Please mark your answers as follows: ☐ ☒ ☐ ☐

Please use a ballpoint pen.

Your responses will not be seen by your treating physician. Please return the questionnaire in the **sealed** envelope provided.

You will not experience any disadvantages if you choose not to participate. In that case, simply return the blank questionnaire in the sealed envelope or discard it.

Thank you for your participation!

Description of Current Health Complaints
(Please mark only one answer per item.)

1. What is the main complaint or reason for your visit today?

Check one reason				Check one location	
Pain	☐	Swelling	☐	Eyeball	☐
Trauma/Injury	☐	Redness	☐	Eyelids	☐
Vision loss	☐	Double vision	☐		

2. According to your own assessment, within what time frame should you receive medical treatment?

Immediately	Within 1 hour	Within 1-2 days	Within 1 week	In 1 month or later
☐	☐	☐	☐	☐

3. How often do your current symptoms occur?

First occurence ☐ Recurrent ☐ Persistent ☐

4. How severe are your current symptoms??

0 = not severe, 10 = extremely severe

0	1	2	3	4	5	6	7	8	9	10
☐	☐	☐	☐	☐	☐	☐	☐	☐	☐	☐

5. How long have your current symptoms been present?

Since today	For several days	For several weeks	For several months
☐	☐	☐	☐

6. How do you rate treatment in the “emergency clinic” compared to a "regular" clinic/practice?

**Emergency clinic**	**worse**	**same**	**better**
Waiting time	☐	☐	☐
Quality of medical care	☐	☐	☐

Overall duration	☐	☐	☐
Diagnostic/Therapeutic options	☐	☐	☐
24/7 availability	☐	☐	☐
Clarification of unclear symptoms	☐	☐	☐

Description of Your Medical Care Situation

7. How many times have you visited an emergency department in the past 6 months (for any medical issue)?

Never ☐ Once ☐ 2-3 times ☐ 4 or more times ☐

8. Do you have a general practitioner (GP)?

Yes ☐ No ☐ Yes, but not in this region ☐

9. Do you have an ophthalmologist?

Yes ☐ No ☐ Yes, but not in this region ☐

10. Did you contact a doctor's office because of your current complaint?
(Contact refers to either visiting or calling a doctor's office.)

Yes ☐ (Continue with section 11A)

No ☐ (Skip to section 11B, continue on the next page)

A: If you contacted a doctor's office

11. Please answer the following questions only if you answered "Yes" to question 10.

1. Were you able to explain your symptoms to the practice team?

   Yes ☐ No ☐

2. Did you have direct contact with a physician (e.g., consultation or examination)?

   Yes ☐ No ☐

3. Was the appointment offered too late in your opinion?

   Yes ☐ No ☐

4. Did the practice recommend that you go to the university hospital?

   Yes ☐ No ☐

B: If you didn’t contact a doctor’s office

11. Please answer the following questions only if you answered "No" to question 10.

1. Do you know the phone number "116117" for the medical on-call service?

   Yes ☐ No ☐

2. Did you try to contact a "regular" practice?

   Yes ☐ No ☐

3. Did you assume that the practice might be closed?

   Yes ☐ No ☐

4. Do you believe a "regular" practice would be sufficient for your current issue?

   Yes ☐ No ☐ Unsure ☐

5. Do you currently have access to a "regular" ophthalmologist?

   Yes ☐ No ☐ Unsure ☐

6. Were you unable to contact a regular clinic due to professional or family reasons?

   Yes ☐ No ☐

7. Are you highly concerned about your symptoms?

   Yes ☐ No ☐

8. Are you visiting the university hospital because you were treated here previously?

   Yes ☐ No ☐

Demographic Information

12. Please indicate your age and gender:

Age (in years): ___________ Gender (male/female/other) ______

To be completed by the physician

Do not open the envelope!

Please mark as follows: ☐ ☒ ☐ ☐

1. How urgent was the treatment?

☐ elective	(sufficient within months)
☐ urgent	(sufficient within weeks)
☐ highly urgent	(sufficient within a few days)
☐ acute	(had to be treated today)
☐ Emergency	(had to be treated immediately, no waiting time)

2. Please rate the following parameters:

**Pain**	**Redness**	**Vision loss**	**Risk of globe rupture**
None ☐	None ☐	None ☐	None ☐
Moderate ☐	Moderate ☐	Moderate ☐	Moderate ☐
Severe ☐	Severe ☐	Severe ☐	Severe ☐

3. Time of examination

1. Weekend

   ☐ Yes ☐ No

2. After 10 p.m.

   ☐ Yes ☐ No
